# A rare case of plexiform neurofibroma

**DOI:** 10.1016/j.radcr.2025.12.021

**Published:** 2026-01-17

**Authors:** Eppy Buchori AK, Ilham Ansari Marzuki Lubis, Radinda Amalia Nur Hayati

**Affiliations:** Department of Radiology RSUP Dr. Hasan SadikinJl. Pasteur No. 38, Bandung, 40161, Indonesia

**Keywords:** Plexiform neurofibroma, MRI, Neurofibromatosis

## Abstract

Plexiform neurofibroma is a rare benign peripheral nerve sheath tumor that occurs almost exclusively in patients with neurofibromatosis type I (NF1). This report presents a 10-year-old patient with multiple enlarging masses on the left and posterior neck for 4 years, accompanied by pain and restricted neck movement. Radiographic evaluation revealed a soft tissue mass without bony abnormalities, while Magnetic Resonance Imaging (MRI) demonstrated multiple confluent, ill-defined, infiltrative lesions involving bilateral cervical regions and extending into the anterior mediastinum. The lesions showed hypointense signals on T1-weighted and hyperintense target sign appearances on T2-weighted images, with encasement of neurovascular structures and spinal cord compression. Histopathological findings confirmed the diagnosis of plexiform neurofibroma without evidence of malignancy. This case emphasizes the diagnostic value of MRI in delineating lesion extent and neurogenic origin, as well as the importance of histopathology in confirming the benign nature of the tumor and excluding malignant transformation.

## Introduction

Peripheral nerve sheath tumors such as neurofibromas account for about 5% of all benign soft tissue neoplasms. A study in Germany stated that the prevalence of NF1 is estimated to be approximately 1 in 3000 individuals, with no association to race. These tumors may appear as solitary or multiple lesions, with most cases associated with neurofibromatosis type I (NF1), which results from mutations in the NF1 gene. The plexiform neurofibroma, a distinctive subtype seen exclusively in NF1, represents an uncommon benign form that develops through disorganized proliferation of all neural components within a peripheral nerve. Histologically, it is characterized by an expanded endoneuria matrix, separation of nerve fascicles, and marked Schwann cell proliferation. Neurofibromas most often present as localized lesions, less commonly in a diffuse form, and only rarely as the plexiform type [1,2].

This case will discuss the Magnetic Resonance Imaging (MRI) findings in a 10-year-old patient with plexiform neurofibroma.

## Case report

### Case presentation

**Fig. 1:**
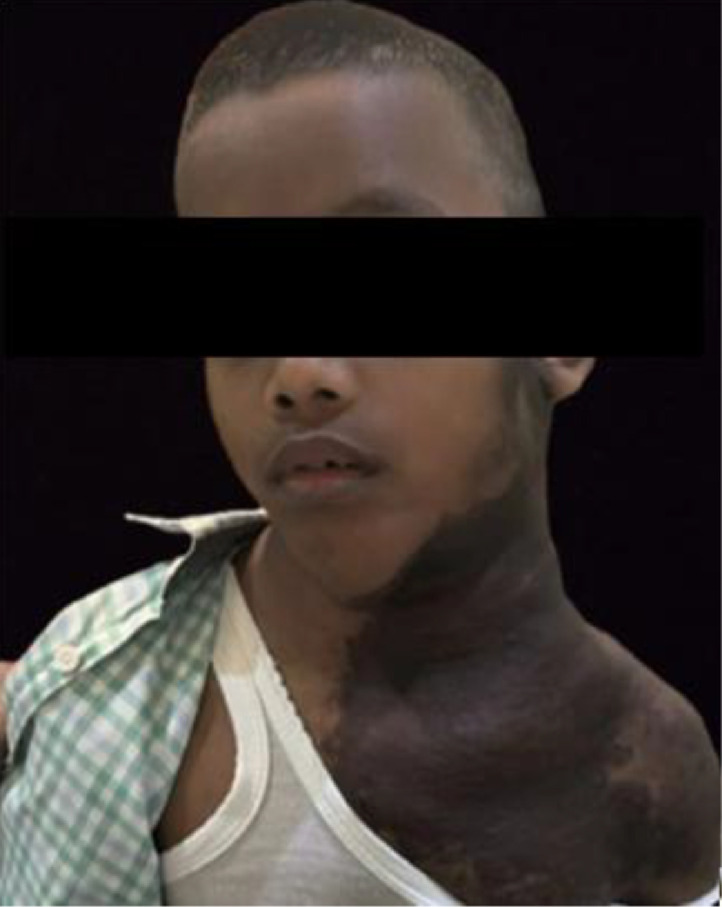
Clinical image of the patient with plexiform neurofibroma.

A 10-year-old patient presented with complaints of lumps on the left side and posterior aspect of the neck that had been present for the past 4 years. The lumps were initially small and multiple, gradually increasing in size over time. The symptoms were accompanied by pain and difficulty turning the head to the left. The patient’s skin also appeared hyperpigmented (Fig. 1). The patient has no visual complaints. There is no family history of the same condition.

### Diagnostic evaluation

**Fig. 2:**
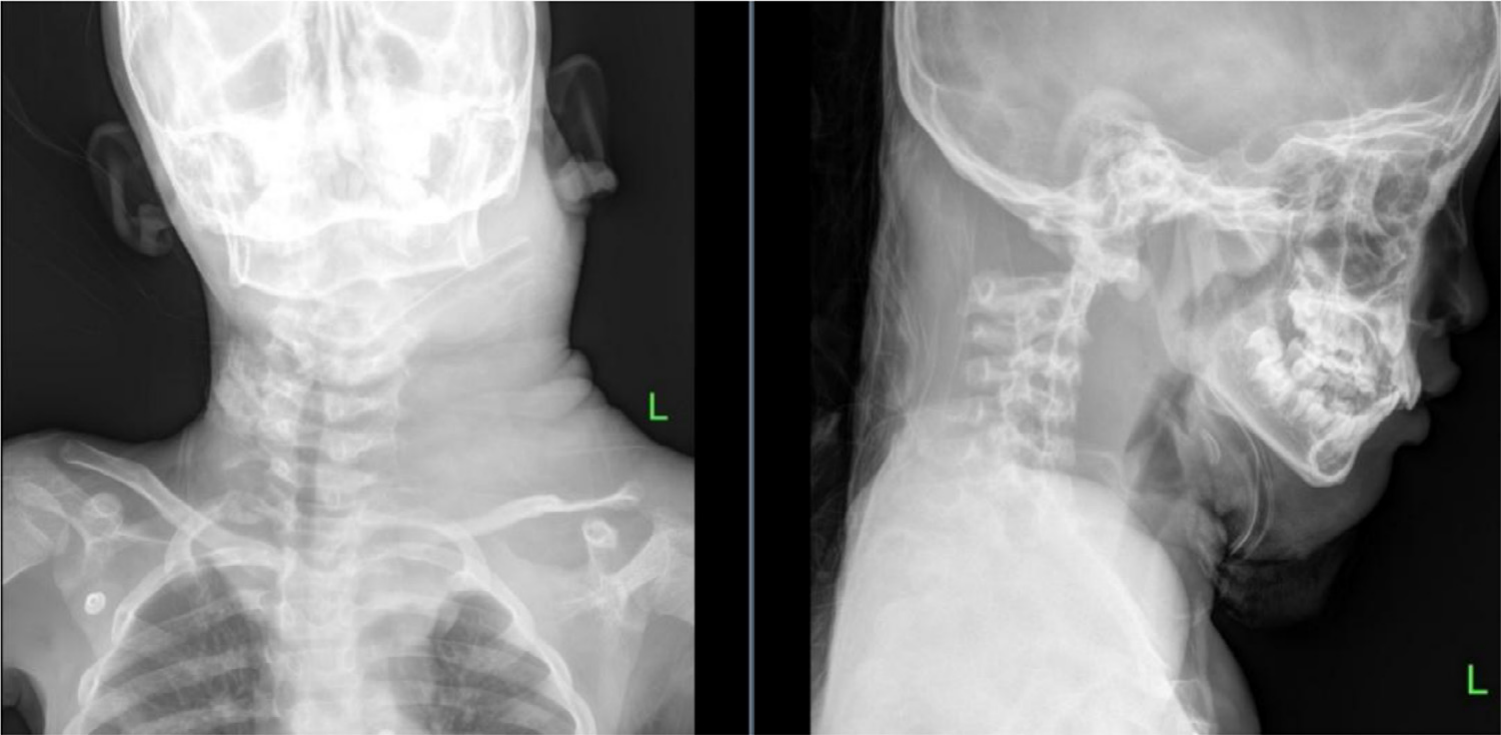
Cervical x-ray of the patient with plexiform neurofibroma.

Initially, a neck X-ray was performed for screening, revealing cervical vertebrae within normal limits. However, a soft tissue density mass without calcification was observed in the left cervical region, causing narrowing of the airway column at the cervical 2-4 level. The retropharyngeal and retrolaryngeal spaces appeared widened (Fig. 2).

**Fig. 3:**
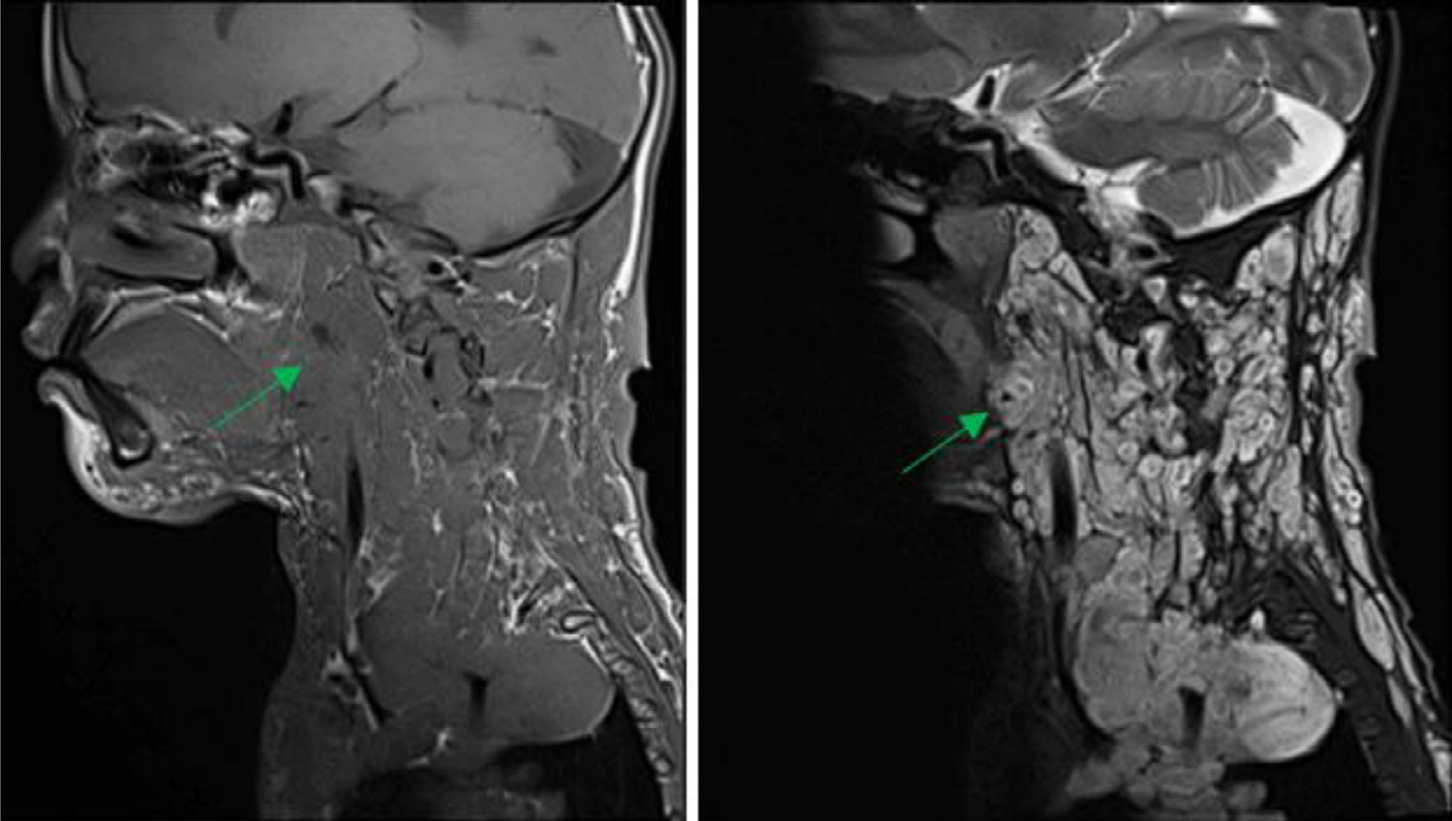
Cervical MRI of the patient with plexiform neurofibroma. (Left) hypointense signal on T1-weighted imaging; (Right) hyperintense signal on T2-weighted imaging.

**Fig. 4:**
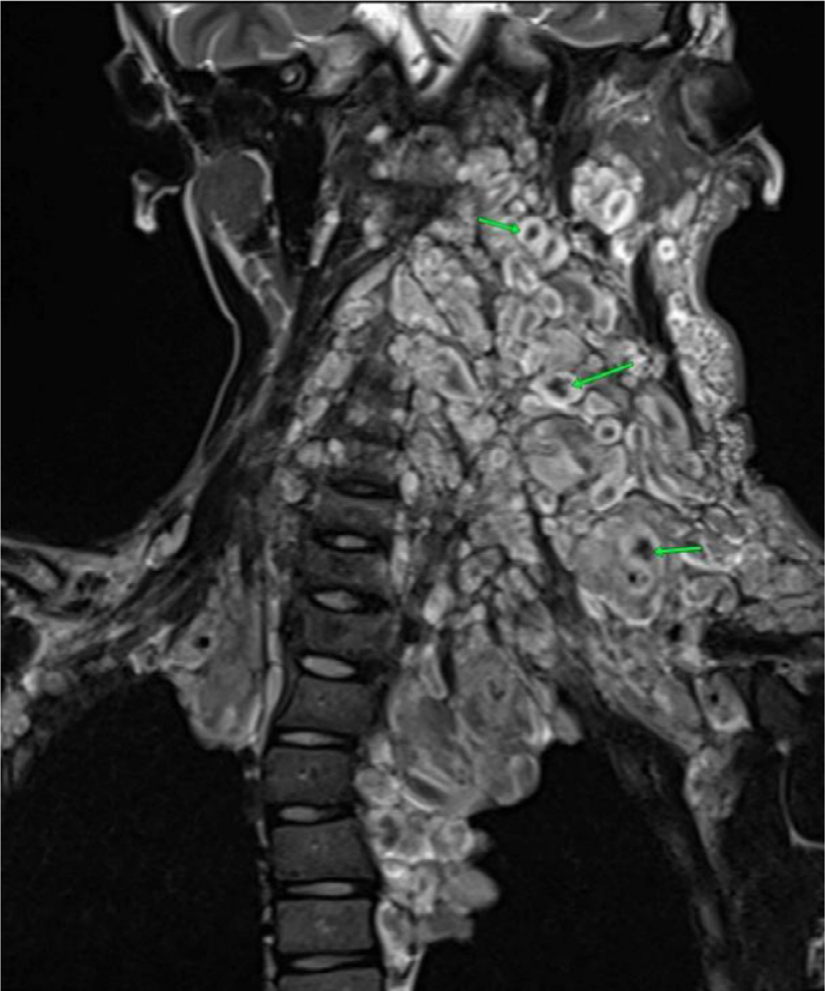
Cervical MRI of the patient with plexiform neurofibromaTarget sign on T2 weight image.

**Fig. 5:**
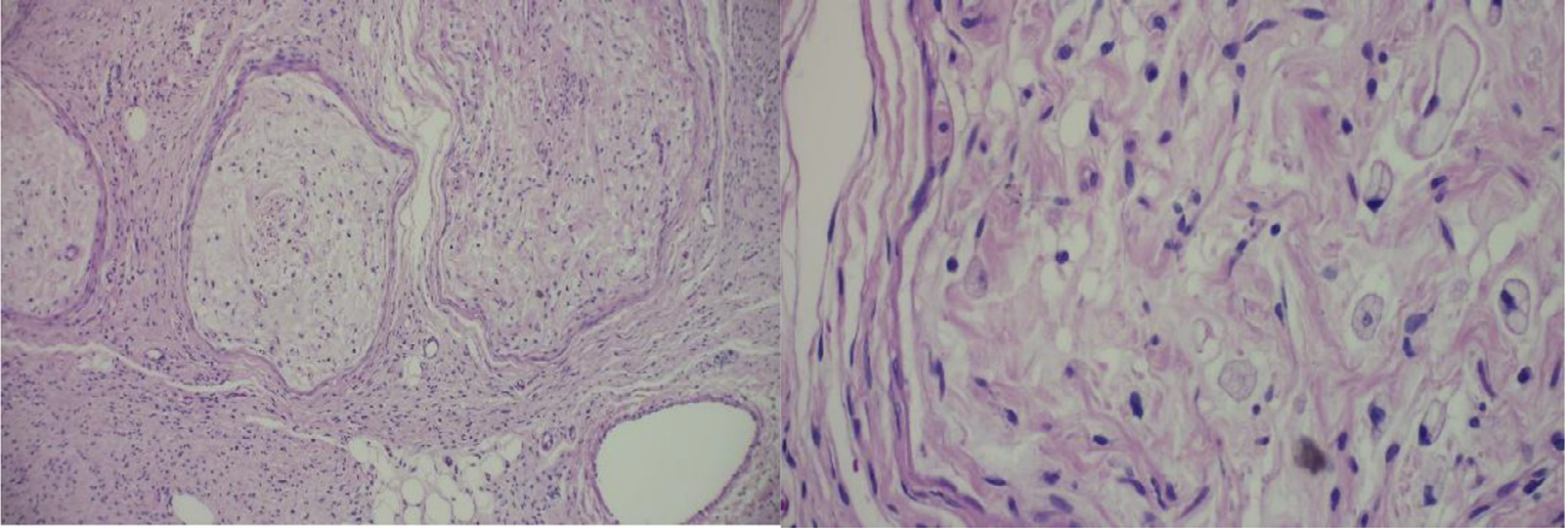
Histopathology of plexiform neurofibroma Spindle-shaped cells showing hyperplastic growth, forming dense clusters with some scattered distribution. The nuclei are partly wavy in appearance, and no mitotic figures are observed. Tumor cells are seen involving nerve fibers surrounded by perineurium.

The patient subsequently underwent a cervical noncontrast MRI, to assess the extent of the mass, which revealed multiple confluent lesions with ill-defined margins and irregular borders in the bilateral cervical regions, more prominent on the left side, extending into the anterior mediastinum. The lesions obliterated the left trapezius, platysma, left sternocleidomastoid, bilateral scalenes, deep cervical paraspinals, and bilateral prevertebral muscles. The mass encased the bilateral common carotid arteries, bilateral subclavian arteries, bilateral internal jugular veins, left external jugular vein, and bilateral vertebral arteries. The lesion demonstrated hypointense signal on T1-weighted images and showed a hyperintense target sign appearance on T2-weighted images (Figs. 3 and 4). The cervical vertebrae appeared unremarkable. Histopathological examination confirmed the diagnosis of plexiform neurofibroma (Fig. 5). The patient has not undergone genetic testing. Follow-up MRI is also required if the lesion continues to enlarge.

## Discussion

Plexiform neurofibromas are intricate lesions, either deep-seated or superficial, originating from peripheral nerve sheaths and present in approximately 40%-50% of individuals with NF1. Clinically, they manifest as firm, thickened masses or nodular enlargements that can infiltrate surrounding tissues extensively, often leading to deformity and functional impairment [3].

Plexiform neurofibromas exhibit a tortuous growth pattern along an extended segment of a peripheral nerve, whereas the plexiform subtype—the rarest form—demonstrates poorly defined, infiltrative expansion. These plexiform lesions are considered virtually pathognomonic for neurofibromatosis type I (NF1) and are found in approximately 40%-50% of affected patients. Histologically, they consist of a proliferation of Schwann cells, spindle cells, mast cells, and vascular components. Due to their deep infiltration, these complex tumors often cause cosmetic deformities and organ dysfunction, rendering complete surgical removal difficult.

On imaging, plexiform neurofibromas typically appear as confluent, multinodular masses that exert a mass effect and may cause adjacent bony erosion. They frequently display multiple target signs on T2-weighted MRI. Compared with localized or diffuse types, plexiform neurofibromas have a higher potential for malignant transformation. Imaging characteristics suggestive of malignancy include large lesion size, heterogeneous signal intensity on T1-weighted sequences, peripheral enhancement, cystic degeneration, and a surrounding edema-like zone [4]. The presence of a target sign on T2-weighted MRI also can aid in distinguishing neurofibromas from malignant peripheral nerve sheath tumors [5].

A case of 41-year-old Asian man with NF1 who presented with bilateral sciatic plexiform neurofibromas. MRI of the left thigh demonstrated several concerning features suggestive of malignant transformation, including a large posterior thigh mass with marked internal heterogeneity and irregular enhancement. The mass also showed thick peripheral enhancement with perilesional edema along its inferior aspect. In contrast, a T2-hyperintense, uniformly enhancing subcutaneous nodule in the left medial thigh corresponded to a previously known benign neurofibroma. These findings highlight the important role of MRI in distinguishing benign from malignant lesions [6].

Plexiform neurofibromas are primarily diagnosed based on their characteristic clinical features, while histopathological examination serves to rule out malignant transformation. MRI serves as the most valuable imaging modality for assessing the extent of the tumor and indicating its neurogenic origin, owing to its superior contrast resolution and multiplanar imaging capabilities. In this patient, the x-ray findings supported the presence of a soft tissue mass. The MRI features were consistent with a plexiform neurofibroma, further confirmed by histopathological examination. No evidence of malignancy was observed [7,8].

Management of plexiform neurofibromas involves an individualized approach based on the tumor’s size, location, growth rate, and associated symptoms. Intervention is generally recommended when the lesion causes functional impairment, pain, cosmetic deformity, or poses a risk of complications, whereas asymptomatic tumors may be observed with routine surveillance. In recent years, targeted therapy with selumetinib has emerged as an effective alternative for inoperable plexiform neurofibroma, with clinical studies demonstrating significant tumor shrinkage, pain reduction, and functional improvement [9].

## Conclusion

Plexiform neurofibroma represents a distinctive benign peripheral nerve sheath tumor that often poses diagnostic and therapeutic challenges due to its infiltrative nature and close association with vital neurovascular structures. MRI plays a crucial role in characterizing the lesion’s extent, internal architecture, and neurogenic origin, while histopathological analysis remains essential for definitive diagnosis and exclusion of malignancy. Early recognition and comprehensive imaging evaluation are vital to guide management and prevent complications related to compression or functional impairment.

## Patient consent

This is to state that I give my full permission for the publication, reproduction, broadcast and other use of photographs, recordings and other audio visual material of myself (including of my face) and textual material (case histories) in all editions of the above named product and in any other publication (including books, journals, CD ROMs, online and internet), as well as in any advertising or promotional material for such product or publications.

I declare, in consequence of granting this permission, that I have no claim on ground of breach of confidence or any other ground in any legal system against – and its agents, publishers, successors a nd assigns in respect of such use of the photograph(s) and textual material (case histories).

I hereby agree to release and discharge, and any editors or other contributors and their agents, publishers, successors and assigns from any and all claims, deman ds or causes of action that I may now have or may hereafter have for libel, defamation, invasion of privacy, copyright or moral rights or violation of any other rights arising out of or relating to any use of my image or case history.
